# Obesity, not a high fat, high sucrose diet alone, induced glucose intolerance and cardiac dysfunction during pregnancy and postpartum

**DOI:** 10.1038/s41598-021-97336-x

**Published:** 2021-09-10

**Authors:** Eunhee Chung, Kassandra Gonzalez, Sarah L. Ullevig, John Zhang, Masataka Umeda

**Affiliations:** 1 Department of Kinesiology, University of Texas at San Antonio, One UTSA Circle, San Antonio, TX 78249 USA; 2 College for Health, Community and Policy, University of Texas at San Antonio, San Antonio, TX USA

**Keywords:** Cardiac hypertrophy, Metabolic syndrome, Obesity, Pre-diabetes

## Abstract

Cardiovascular disease is the leading cause of death in women during pregnancy and the postpartum period. Obesity is an independent risk factor for cardiovascular diseases. Nearly 60% of women of reproductive age are considered overweight or obese, cardiovascular disease morbidity and mortality continue to be pervasive. The objective of this study was to determine the effects of an obesogenic diet on the cardiometabolic health of dams during pregnancy and postpartum. Female mice were fed either a high-fat, high-sucrose diet (HFHS) or a refined control diet (CON) for 8 weeks before initiation of pregnancy and throughout the study period. Mice in the HFHS showed two distinct phenotypes, obesity-prone (HFHS/OP) and obesity resistance (HFHS/OR). Pre-pregnancy obesity (HFHS/OP) induced glucose intolerance before pregnancy and during postpartum. Systolic function indicated by the percent fractional shortening (%FS) was significantly decreased in the HFHS/OP at late pregnancy (vs. HFHS/OR) and weaning (vs. CON), but no differences were found at 6 weeks of postpartum among groups. No induction of pathological cardiac hypertrophy markers was found during postpartum. Plasma adiponectin was decreased while total cholesterol was increased in the HFHS/OP. Our results suggested that obesity, not the diet alone, negatively affected cardiac adaptation during pregnancy and postpartum.

## Introduction

Cardiovascular disease is the leading cause of death in women during pregnancy and the postpartum period. Nearly 60% of women of reproductive age are considered overweight or obese, which contributes to the rise in cardiovascular disease among this population. Pregnancy is known to induce cardiac hypertrophy as seen by increases in left ventricular mass, end-diastolic diameters, and wall-thickness when compared to non-pregnant controls^1–3^. Pregnancy-induced cardiac hypertrophy serves as an adaptive response as it enables the heart to increase its contractile power and to minimize the wall stress to meet maternal and fetal growth^4^. Systolic function indicated by the percent fractional shortening (%FS) or the percent ejection fraction (%EF) is well preserved during most of the pregnancy but decreases near term^1,3^ and early postpartum^5,6^, possibly due to preload reduction^6^. Moreover, impaired myocardial relaxation with diastolic dysfunction at term suggests cardiovascular maladaptation could occur in some normal pregnancies^7^.

The prevalence of cardiac hypertrophy associated with volume overload is common in people with obesity that can lead to cardiac dysfunction^8^. Emerging evidence suggests that pre-pregnancy obesity and greater weight gain in early pregnancy are highly associated with maternal complications^9,10^. Thus, obesity could negatively affect the maternal heart, which already undergoes cardiac adaptation in response to normal pregnancy^7^. However, there is a lack of studies examining how pre-pregnancy obesity and early gestational weight gain influence maternal cardiac adaptation to the increasing cardiovascular demands of pregnancy, lactation, and following pregnancy with persistent nutritional challenges. Therefore, the objective of this study was to evaluate cardiac adaptation at term, at weaning, and postpartum in response to an obesogenic diet. We hypothesized that maternal obesity would challenge pregnancy-induced cardiac adaptation during pregnancy and persistent dietary challenges would be detrimental to the cardiometabolic health of postpartum female mice.

## Results

### Morphological characteristics

**Figure 1:**
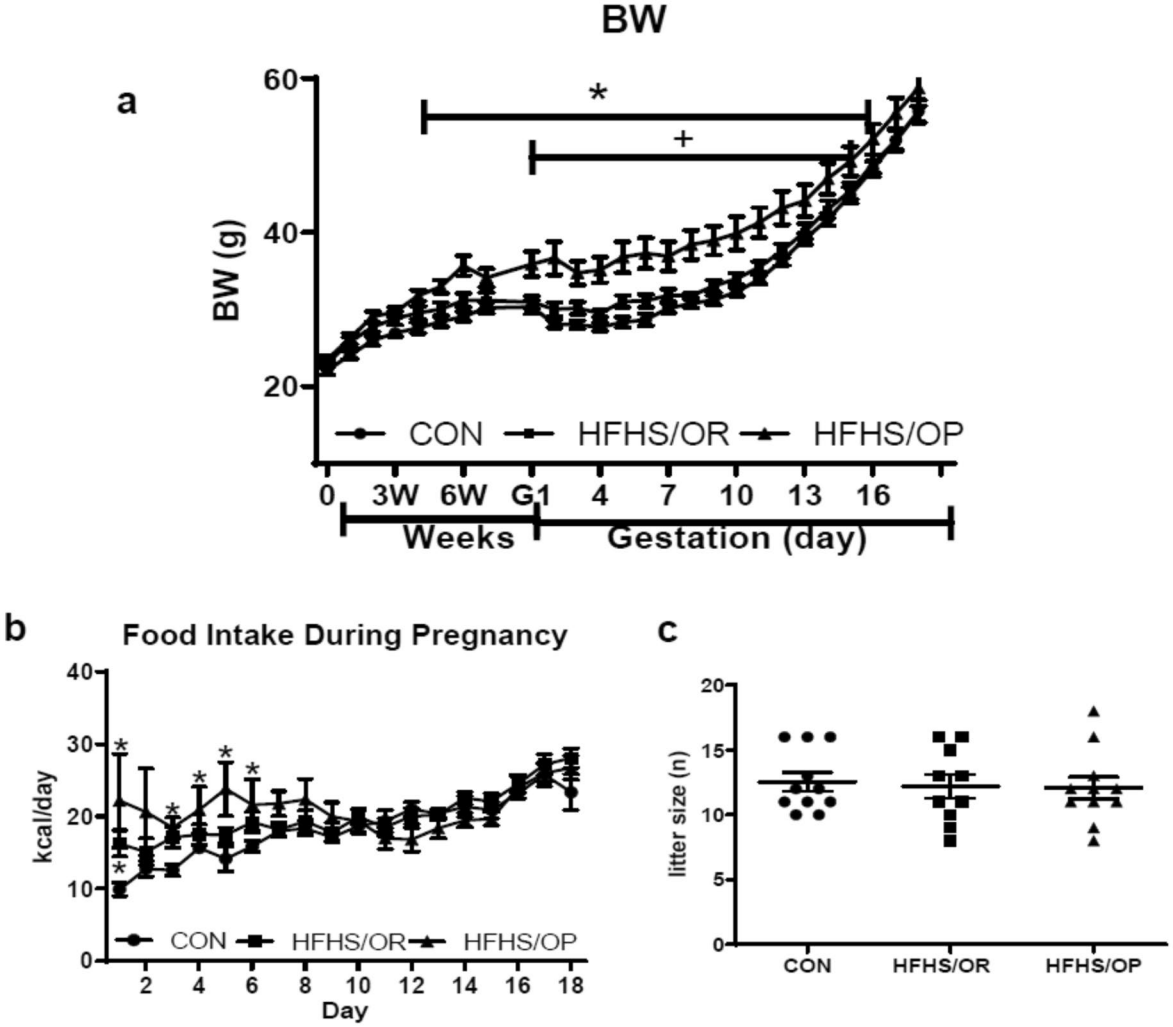
The effects of diet on body weight, food intake, and litter size. (**a**) Body weight (BW) increased over time with the HFHS group weighing significantly more than the CON group from the fourth to eighth week of diet intervention and much greater in the HFHS/OP group than the HFHS/OR group. (**b**) Food intake was significantly higher in HFHS/OR compared to the CON at day 1 of gestation, and in HFHS/OP compared to the CON until gestational day 6. As pregnancy progresses (gestational day 7–18), no group differences were observed. (**c**) litter sizes were not different among groups. CON, n = 11; HFHS/OR, n = 10, and HFHS/OP, n = 11. CON, control diet indicated by closed circles (filled circle); HFHS/OR, obese-resistant mice fed with a high-fat and a high-sucrose diet indicated by closed squares (filled square); HFHS/OP, obese prone mice fed a high-fat high-sucrose diet indicated by a closed triangle (filled triangle); Values are presented as mean ± SEM. Statistical significance is calculated by two-way ANOVA followed by Tukey’s multiple comparisons (**a** and **b**) or one-way ANOVA (**c**). *p < 0.05, versus CON; + *p* < 0.05, versus HFHS/OR.

While initial body weight (BW) was similar, we found significant differences in weight gain after diet intervention, but with divergent phenotypes in the HFHS-fed mice resulting in obese-prone (HFHS/OP) and obese resistance (HFHS/OR) mice. Mice in the HFHS/OP group had significant weight gain as compared to the HFHS/OR and CON groups (Table 1). A significant weight gain was observed after 4 weeks of diet intervention in the HFHS/OP group until gestational day 16 compared to CON (Fig. 1a). Mice in the HFHS/OP were also significantly heavier after 6 weeks of diet intervention until gestation day 14 than the HFHS/OR (Fig. 1a). No BW differences were found between CON and HFHS/OR before gestation, during gestation, and 8 weeks of postpartum (Table 1). Although %BW gain before gestation was significantly higher in HFHS/OP compared to HFHS/OR or CON, gestational weight gain was not significantly different among groups (Table 1). The calorie intake was significantly higher in the HFHS/OP compared to the HFHS/OR and CON groups until gestational day 6, but as gestation progresses, the caloric intake among groups was not different (Fig. 1b). As expected, postpartum body weight (i.e., final BW) was much higher in the HFHS/OP group with continuous feeding of HFHS, but no differences were found between CON and HFHS/OR groups. Neither sex distribution (Supplementary Figure 1) nor the litter size (Fig. 1c) were affected by maternal diet or the status of obesity.

**Table 1:** General characteristics of dams.

Measurement	CON (n = 11)	HFHS/OR (n = 10)	HFHS/OP (n = 11)
Initial BW (g)	22.08 ± 0.57	23.00 ± 0.54	23.18 ± 0.57
% WG before gestation	23.03 ± 3.40	19.33 ± 1.85	38.21 ± 4.96*^+^
% WG during gestation	44.59 ± 2.08	43.50 ± 1.76	39.71 ± 2.42
Final BW (g)	36.28 ± 1.26	41.62 ± 1.58	46.48 ± 2.19*
HW (mg)	152.90 ± 4.31	159.60 ± 6.61	165.10 ± 4.51
HW/BW (mg/g)	4.23 ± 0.10	3.86 ± 0.15	3.59 ± 0.11*
HW/TL (mg/mm)	7.84 ± 0.20	8.10 ± 0.34	8.44 ± 0.23

Values are expressed as mean ± SEM. CON, control; HFHS/OR, obese-resistant mice fed a high-fat, high-sucrose diet; HFHS/OP, obese-prone mice fed a high-fat, high-sucrose; BW, body weight; HW, heart weight; TL, tibial length; WG, weight gain. *P < 0.05 versus CON, ^**+**^*P* < 0.05 versus HFHS/OR.

### Glucose- and insulin tolerance tests

**Figure 2:**
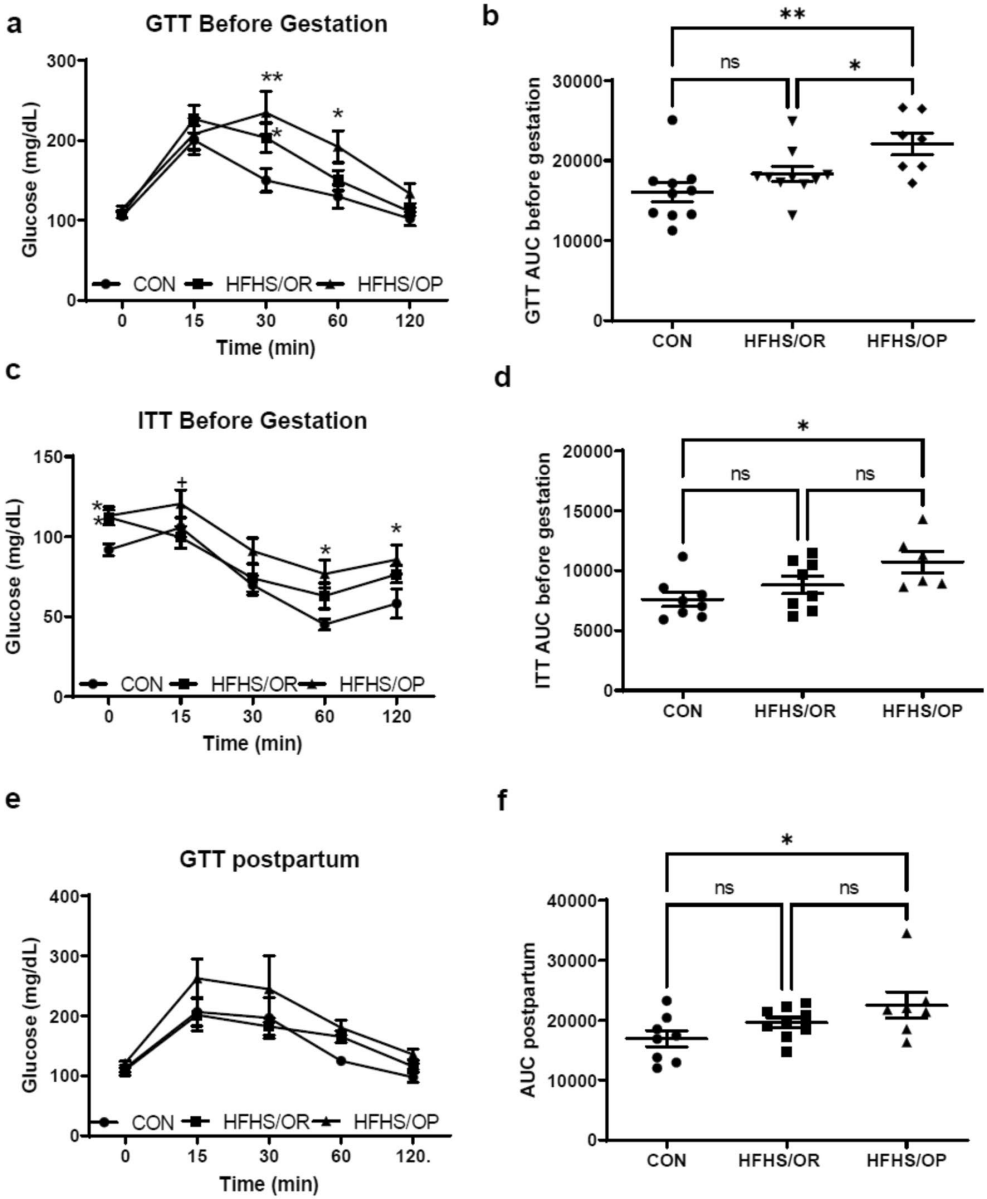
Glucose and insulin tolerance tests. GTT and ITT measurements at 0, 15, 30, 60, and 120 min after glucose (**a** and **e**) and insulin injection (**c**). (**a**) The HFHS/OP group had significantly impaired glucose clearance and insulin-stimulated glucose clearance compared to the CON group. The area under the curve (AUC) of GTT (**b**) and ITT (**d**). Impaired glucose clearance (**e** and **f**) was persistent at 6 weeks of postpartum. CON, control diet indicated by closed circles (filled circle); HFHS/OR, obese-resistant mice fed with a high-fat and a high-sucrose diet indicated by closed squares (filled square); HFHS/OP, obese prone mice fed a high-fat high-sucrose diet indicated by a closed triangle (filled triangle). An individual dot indicates the sample size; n = 6–10/group. Values are presented as mean ± SEM. Statistical significance is calculated by two-way ANOVA followed by Tukey’s multiple comparisons (**a**, **c**, and **e**) or one-way ANOVA (**b**, **e**, and **f**).* *p* < 0.05, versus CON; + *p* < 0.05, versus HFHS/OR; ** *p* < 0.001, versus CON.

Compared with the CON group, the HFHS/OP group showed impaired glucose tolerance after 8 weeks of diet intervention (Fig. 2a). The mice in the HFFS/OP had worse glucose tolerance than the mice in the HFHS/OR at 30 min and 60 min following glucose injection. Glucose tolerance test (GTT) area under the curve (AUC) before gestation (Fig. 2b) was significantly greater in HFHS/OP when compared to both CON and HFHS/OR. For the insulin tolerance test (ITT), the mice in the HFHS/OP and HFHS/OR had higher fasting glucose levels and developed insulin resistance as shown by a slower decrease in blood glucose levels at 60 and 120 min after insulin injection compared to the CON. The mice in the HFHS/OP had much higher blood glucose at 15 min after insulin injection compared to the CON and the HFHS/OR (Fig. 2c). The difference in ITT AUG between HFHS/OP and CON reached a statistical significance, but no differences were found between the CON and the HFHS/OR as well as between the HFHS/OR and HFHS/OP (Fig. 2d). Impaired glucose tolerance before pregnancy (Fig. 2a, b) was persistent at 6 weeks postpartum in HFHS/OP as compared to control mice, which was well demonstrated in GTT AUC (Fig. 2f), but not at each time point (Fig. 2e).

### Plasma glucose, lipid biomarkers, and hormones

Fasting blood glucose levels were not different among groups (Table 2). Lipid biomarkers, including triglyceride (TG), non-esterified free fatty acid, low-density lipoprotein (LDL), and high-density lipoprotein (HDL) were not significantly different among groups (Table 2). However, total cholesterol (TC) was significantly higher in the HFHS/OP than in the CON (Table 2). Insulin, leptin, and resistin were not different among groups. Nevertheless, the mice in the HFHS/OP group had substantially lower adiponectin levels than the HFHS/OR (Table 2).

**Table 2:** Measured plasma glucose, lipids, and hormones measured at 8 weeks postpartum.

	CON	HFHS/OR	HFHS/OP
Fasting blood glucose (mg/dL)	140.2 ± 16.8 (10)	138.3 ± 12.9 (10)	173.0 ± 15.6 (11)
Triglycerides (mg/dL)	52.5 ± 10.3 (6)	65.29 ± 8.1 (7)	66.43 ± 12.9 (7)
Free fatty acids (μM)	432.2 ± 42.4 (6)	581.3 ± 112.1 (6)	468.1 ± 100.7 (7)
Total cholesterol (mg/dL)	110.2 ± 6.0 (6)	119.6 ± 9.9 (7)	160.0 ± 15.9* (7)
LDL (mg/dL)	28.5 ± 6.5 (6)	31.29 ± 4.9 (7)	49.7 ± 8.1 (7)
HDL (mg/dL)	104.8 ± 7.8 (6)	108.3 ± 6.9 (7)	100.6 ± 7.2 (7)
Insulin (ng/ml)	1.02 ± 0.34 (5)	1.03 ± 0.49 (3)	1.70 ± 0.32 (7)
Leptin (ng/ml)	0.91 ± 0.26 (5)	7.44 ± 3.93 (3)	6.78 ± 3.88 (7)
Resistin (ng/ml)	127.6 ± 39.7 (5)	122.4 ± 52.8 (3)	108.5 ± 18.9 (7)
Adiponectin (ng/ml)	7888.0 ± 529.4 (7)	8851.0 ± 926.4 (7)	6557.0 ± 281.9^+^ (8)

Values are expressed as mean ± SEM; Parenthesis indicates the sample size. CON, control; HFHS/OR, obese-resistant mice fed a high-fat high-sucrose diet; HFHS/OP, obese-prone mice fed a high-fat, high-sucrose; LDL, low-density lipoprotein; HDL, high-density lipoprotein; *P < 0.05 versus CON, ^**+**^*P* < 0.05 versus HFHS/OR.

### Functional and molecular profiles of the heart

**Figure 3:**
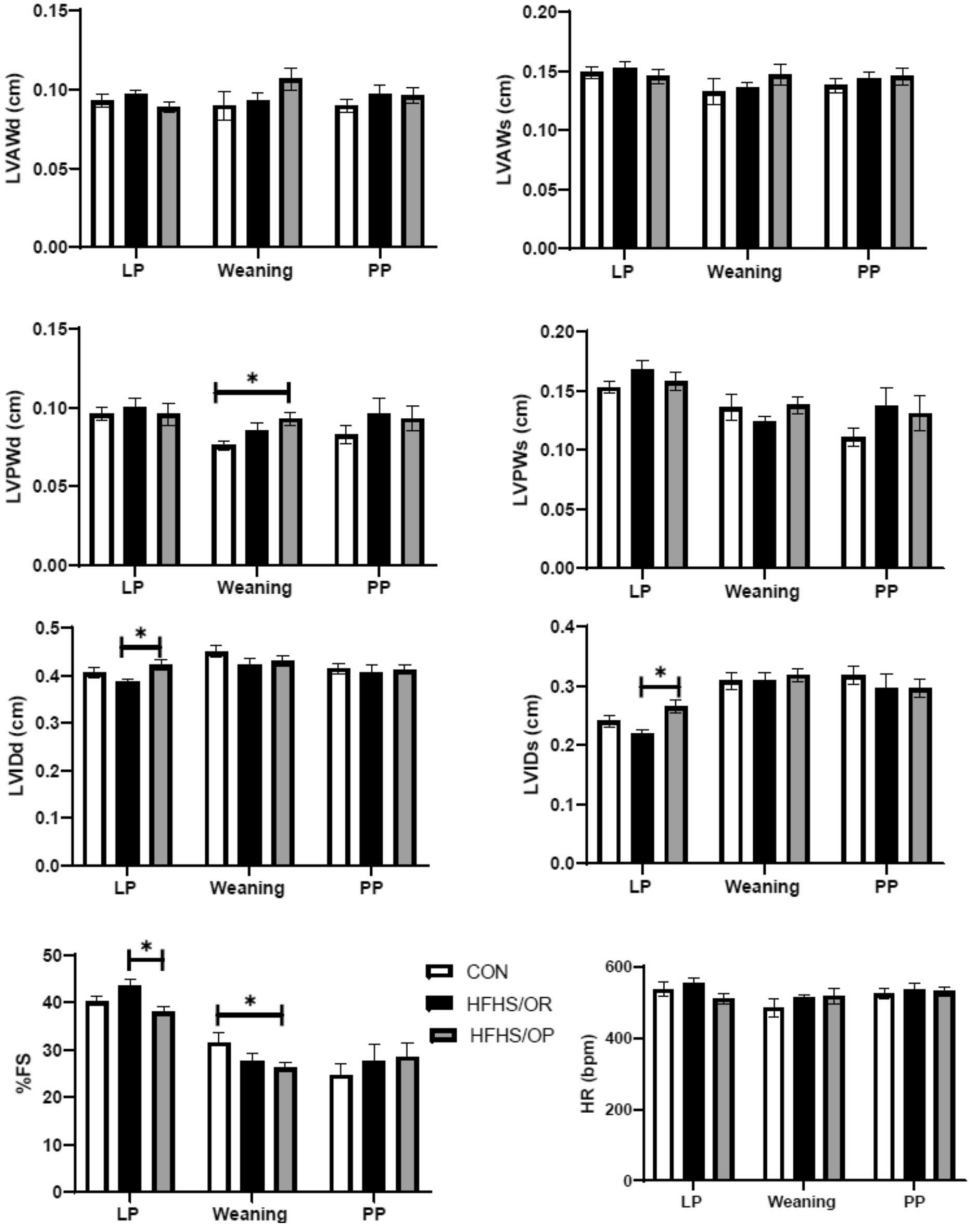
Cardiac function measured by M-mode Echocardiography. Cardiac function was not altered by the diet or obesity during postpartum although decreased cardiac function was observed at LP and weaning. Echocardiography was done at 1 to 2 days before parturition (LP), at weaning (21 days after delivery), and 6 weeks postpartum. CON, control diet; HFHS, obese-resistant mice fed with a high-fat and a high-sucrose diet; HFHS/OP, obese prone mice fed a high-fat high-sucrose diet. The data were shown together for easy to acquire and not for the comparison among different measurement phases. Values are expressed as mean ± SEM; n = 7–11/group. Statistical significance is calculated by one-way ANOVA. LVAWd, left ventricular anterior wall at diastole; LVIDd, left ventricular interior dimension at diastole; LVPWd, left ventricular posterior wall at diastole; LVAWs, left ventricular anterior wall at systole; LVIDs, left ventricular interior dimension at systole; LVPWs, left ventricular posterior wall at systole; % FS, percent fractional shortening. CON, white bars; HFHS/OR, black bars; HFHS/OP, gray bars.

Diet or obesity status did not affect the cardiac mass: the absolute heart weight (HW) and relative HW (HW normalized by tibial length) were not significantly different among groups (Table 1). Significantly lower HW/BW in the HFHS and HFHS/OP groups than the CON was largely due to higher final body weight in the mice fed HFHS. Figure 3 showed the cardiac function measured by echocardiography at late gestation, at weaning, and 6 weeks of postpartum. The left ventricular chamber size, indicated by interior dimension (LVID) was significantly greater in HFHS/OP when compared to HFHS/OR during both systole and diastole at late pregnancy. In addition, the percent fractional shortening (%FS) was shown to be significantly lower in HFHS/OP when compared to HFHS/OR in late pregnancy. Left ventricular posterior wall thickness (LVPW) during diastole was significantly greater in HFHS/OP when compared to the CON at weaning as well as the %FS. However, no significant differences were found among groups at 6 weeks postpartum.

**Figure 4:**
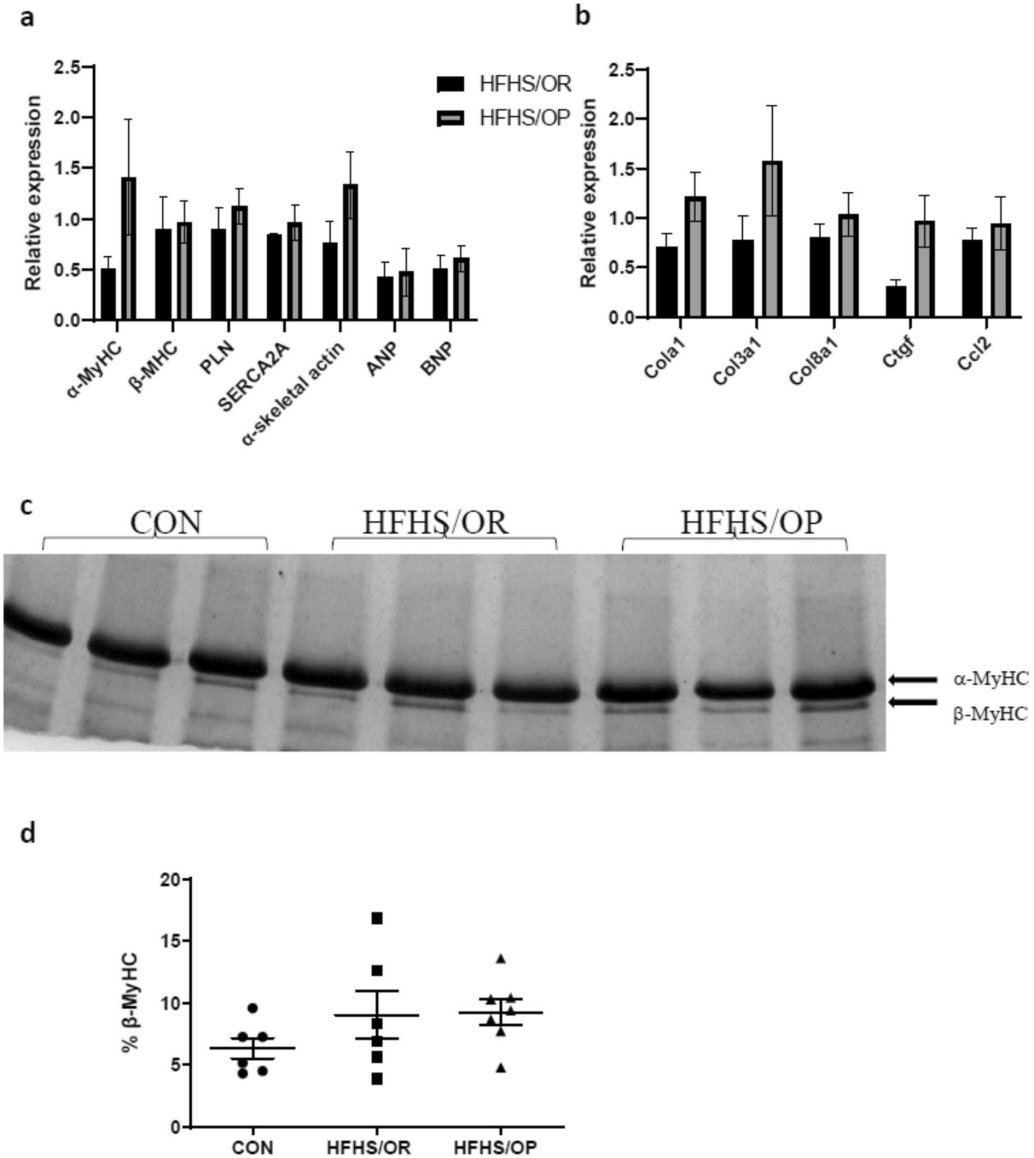
mRNA levels and myosin heavy chain isoform analyses. (**a**,**b**) genes associated with pathological cardiac hypertrophy and interstitial collagen content were not altered by diet or obesity status; n = 4–8/group. CON, white bars; HFHS/OR, black bars; HFHS/OP, gray bars. (**c**) a representative image was obtained from the single gel showing myosin heavy chain (MyHC) isoforms and (**d**) the bar graph showing the % β-MyHC was obtained from 6 to 7 animals per group with 2 to 3 technical replicates per animal. Statistical significance is calculated by one-way ANOVA. CON, control diet indicated by closed circles (filled circle); HFHS/OR, obese-resistant mice fed with a high-fat and a high-sucrose diet indicated by closed squares (filled square); HFHS/OP, obese prone mice fed a high-fat high-sucrose diet indicated by a closed triangle (filled triangle). An individual dot indicates the sample size; n = 6–7/group with two to four technical replicates per animal. ANP, atrial natriuretic peptide; BNP, brain natriuretic peptide; *Ccl2*, C–C motif chemokine ligand 2; *Col1a1*, collagen type 1 alpha chain; *Col3a1*, collagen type III alpha chain; *Col8a1*, collagen type VIII alpha 1 chain; *Ctgf*, connective tissue growth factor; PLN, phospholamban; SERCA2A, sarcoplasmic reticulum Ca^2+^ ATPase 2a.

Pathological cardiac hypertrophy is commonly accompanied by the induction of genes that are predominately expressed in the fetal heart including β-myosin heavy chain (MyHC), phospholamban (PLN), sarcoplasmic reticulum Ca^2+^ ATPase 2a (SERCA2A), α-skeletal actin, atrial natriuretic peptide (ANP), and brain natriuretic peptide (BNP). In addition, increased interstitial fibrosis^4^ is a common characteristic of pathological hypertrophy. Thus, we measured genes associated with pathological cardiac hypertrophy (Fig. 4a) and extracellular matrix (Fig. 4b) in hearts from 8 weeks of postpartum. We found that these genes were not altered by diet or obesity status. Further, we determined MyHC isoforms, which affect the power output properties of the heart, such that increased β-MyHC is highly correlated with lower power output compared to α-MyHC. We found that the percent of α-MyHC or β-MyHC were not altered among groups (Fig. 4c,d).

## Discussion

The primary finding from this study was that pre-pregnancy obesity and early gestational weight gain, not HFHS itself, induced weight retention and glucose intolerance during postpartum that is accompanied by significantly decreased circulating adiponectin levels. Despite postpartum obesity and impaired glucose tolerance, cardiac remodeling during postpartum was not affected by diet or obesity status: neither genes associated with pathological cardiac hypertrophy nor echocardiographic parameters including %FS were not altered at 6 weeks of postpartum.

In this study, we affirmed the previous animal studies^11,12^ fed an obesogenic diet (i.e., HFD) showing the dimorphic phenotypes: obesity-prone or obesity-resistant. Consistent with the human subjects that show lean women generally gain more body weight compared to women that are obese during pregnancy^13^, body weight was similar among groups in late pregnancy largely due to the similar caloric intake as the pregnancy progressed. Moreover, our results agreed with the clinical data^14^ indicating pre-pregnancy obesity and early gestational excess weight gain are associated with long-term postpartum obesity as well as impaired glucose homeostasis. The low levels of adiponectin may explain the exaggerated glucose intolerance and insulin resistance^15–17^ observed in the HFHS/OP. However, HFHS feeding itself shown in HFHS/OR did not alter plasma adiponectin levels, which agreed with previous studies^18,19^. Fasting glucose and plasma insulin levels were not different among groups at 8 weeks postpartum. It is common to see no differences in fasting glucose levels with impaired glucose and insulin tolerance in prediabetes and T2DM^20,21^. It has been suggested that hepatic insulin resistance induced by nonalcoholic fatty liver disease is a major contributor to the transition from normal glucose level to fasting hyperglycemia and T2DM^22,23^.

Increased lipid levels throughout pregnancy are normal in healthy pregnant women to accommodate the nutrient needs of developing fetuses when compared to non-pregnant women^24^. We did not find any differences in lipids including NEFA, LDL, HDL, TG, except TC among groups at 8 weeks postpartum, which agreed with the previous studies^19,25^. However, TC levels were significantly higher in the HFHS/OP than the CON suggesting that obesity status may influence cholesterol metabolism^26,27^. Further, we did not find any differences in resistin and leptin at 8 weeks postpartum, which are typically altered with obesity and T2DM^19,28^. Previous studies have demonstrated the link between adiposity and metabolic hormones in non-pregnant groups^28,29^. However, the results on lipids, inflammatory markers, and hormones in pregnancy are inconsistent due to the different measurement phases (i.e., 1st, 2nd, or third trimester, during lactation, or postpartum). For example, systemic inflammation and obese-linked hormones are higher in obese humans and rodents before pregnancy^30–32^, but these levels are normalized by late gestation^32,33^.

Significantly decreased systolic dysfunction indicated by %FS in HFHS/OP group at late pregnancy and weaning may be attributed to increased left ventricle end-diastolic dimension (LVIDd) or increased posterior wall thickness (LVPWd)^34^. However, decreased %FS was no longer persistent at 6 weeks postpartum which agreed with previous pregnancy studies in mice fed with the standard laboratory chow diet. For example, decreased cardiac function seen in late pregnancy^1,3^, and early postpartum^5^ returns to normal during postpartum^18,35^. Supporting our functional data at postpartum, fetal genes were not induced and genes regulating fibrosis and interstitial collagen content were not altered. Our results contradict several other studies investigating postpartum cardiac remodeling^18,36^. These differing results may be due to the differences in the postpartum phase (1 day postpartum^36^ vs. 6 weeks of postpartum in our study) or lactation status (no lactation by removing pups right after delivery^18^ vs. normal lactation in our study). Indeed, Poole et al., demonstrates that interruption of lactation negatively affects postpartum cardiovascular function^37^, while lactation improved maternal metabolism^38^ even after weaning^39^. Thus, future studies are merited to determine whether the protective effects of lactation or lactation-related hormones may alleviate diet-induced cardiac remodeling during the postpartum period by comparing non-lactating mice.

There are some limitations. Although we reported the cardiac function at different periods of pregnancy (i.e., late pregnancy, at weaning, and 6 weeks of postpartum), we did not compare the echocardiographic parameters over time since two independent experimental animal cohorts were used. Similarly, we did not measure genes regulating pathological cardiac hypertrophy and extracellular matrix at the different phases of pregnancy although we found significantly decreased cardiac function at late pregnancy and at weaning in the HFHS/OP. Future studies are warranted to investigate the mechanisms involved in cardiac dysfunction in the aspects of fuel metabolism, mitochondrial dysfunction independent from systemic contribution (ex., atherosclerotic plague formation along with endothelial dysfunction) over the time course of pregnancy and postpartum.

In summary, we showed that the HFHS/OP mice developed glucose intolerance before pregnancy and at 6 weeks postpartum. Our data reinforce published human studies^40,41^ indicating preventative interventions should be introduced pre-pregnancy and early pregnancy to manage excessive weight gain to reduce risks of developing metabolic disorders in later life. Our results further add to a body of evidence that lifestyle modification is strongly recommended for women with pre-pregnancy obesity and insulin resistance to reduce the risk of developing T2DM later in life.

## Materials and methods

### Diets and experimental groups

The HFHS diet (TD.08811, Envigo, USA) supplies 4.7 kcal/g with 45, 15, and 40% total calories from fat, protein, and carbohydrate, respectively, with 34% sucrose wt:wt. The CON diet (TD.170522, Envigo, USA) supplies 3.8 kcal/g with 17, 18, and 64% total calories from fat, protein, and carbohydrate, respectively with 12% sucrose wt:wt. The detailed diet formula is listed in Supplementary Table 1. The fat composition of our refined control diet was increased to a standard laboratory chow diet (17% total calories from fat) as recommended by the American Institute of Nutrition for Pregnancy^42^.

Five-week-old virgin female Institute for Cancer (ICR) mice with a body weight range of ± 2 g were obtained from Charles River’s Laboratory (Wilmington, USA) and were acclimated in the facility for 7 days on a standard laboratory chow diet (PicoLab Select Rodent 50 IF/6F, 5V5R, Lab Diet, USA). Female mice were group-housed (n = 3–4 per cage). After 1-week of acclimation (at 6 weeks of age), female mice were randomly assigned to a HFHS or a refined control diet (CON). Considering fertility challenges associated with obese animals^43,44^, more animals were assigned to the HFHS group as compared to the CON group. After 4 weeks of diet intervention, half of the HFHS mice gained BW significantly, while the other half were similar in weight gain as the CON mice. Thus, we analyzed the data into three groups: CON, HFHS with similar weight gain as CON before pregnancy (HFHS/OR), and HFHS with the development of obesity before pregnancy (HFHS/OP), and reported the results accordingly. After 8 weeks of diet intervention, breeding was initiated with a harem (3–4 female mice per 1 male mouse) to minimize paternal effects on offspring^45^. The male mouse was added to the female cage when the female mice were in the proestrus or estrus cycle and removed right after confirming pregnancy. Pregnancy was examined the next morning by checking the copulatory plug (day 1 of gestation), and the pregnant dams were individually housed with their respective diets by the end of the study, except during mating when female mice were fed the same diet as the male mouse (standard laboratory chow diet). Body weight was measured weekly from week 1 to week 7 of the diet intervention and daily during gestation days 1 to 18. Food intake was measured daily during gestation. Animals were given ad libitum access to food and water. Supplementary Figure 2 showed the experimental scheme. Two independent experimental animal cohorts were used for all in vivo data. Animal care procedures were approved by the University of Texas at San Antonio Institutional Animal Care and Use Committee, and all experiments were performed following the relevant guidelines and regulations (protocol # MU109). The study was reported under ARRIVE guidelines^46^.

### In vivo glucose- and insulin tolerance tests

Before mating and at 6 to 7 weeks postpartum, an intraperitoneal glucose tolerance test (GTT) was measured 5 h of fasted status using a glucometer (AimStrip Plus, VWR, USA). D-glucose dissolved in phosphate-buffered saline (2 g/kg body weight) was injected intraperitoneally and blood samples (< 5 µl) were collected from the tail vein before injection (basal fasting glucose level) and at 15, 30, 60, and 120 min after the injection. Similar to GTT, insulin tolerance test (ITT) was performed before mating on 5 h fasted animals and blood glucose levels were measured immediately before injection of 1U/kg body weight of insulin (Humulin R, 100U/ml, Henry Schein Animal Health, USA) intraperitoneally, and at 15, 30, 60, and 120 min after injection. A 1-week wash-out period was given between GTT and ITT to reduce the stress of the animals. We attempted ITT during postpartum, but most mice in the CON group underwent hypoglycemia and required glucose injection. The total area under the curve (AUC) was calculated by the trapezoidal method.

### Cardiac function assessment

Left ventricular systolic function and dimensions were measured at the midpapillary short-axis views by M-mode echocardiography using the high-resolution NEXTGen LOGIQ e R7 (GE, USA) ultrasound system equipped with a 10–22 MHz transducer. Mice were sedated with isoflurane (2.5% induction and 1.5% maintenance) and measured in a supine position on a warming pad. Echocardiography was performed at late gestation (1–2 days before parturition), at weaning, and at 6 weeks postpartum, and evaluated separately since two independent experimental animal cohorts were used. However, the data were shown together for easy to acquire and not for the comparison among different measurement phases.

### Blood and tissue collection

The dams were studied 8 weeks postpartum (26–28 weeks old), about 20–22 weeks on their diets to avoid transient changes of hormones and lipids associated with pregnancy. Following 5 h of fasting, the blood samples were collected by cardiac puncture from mice anesthetized with isoflurane and placed in tubes containing ethylenediaminetetraacetic acid (EDTA, final concentration 5 mM), and then centrifuged (1500 × *g*, 15 min at 4 °C) for plasma collection. Plasma was stored at − 80 °C for further analysis. Following blood collection, mice were euthanized by cervical decapitation after the inhalation of a high dose of isoflurane. Hearts were rapidly excised and washed in phosphate-buffered saline (PBS) to allow blood to be pumped out of the cardiac chambers and coronary vessels. The hearts were dried on blotting paper, weighed, frozen in liquid nitrogen, and stored at − 80 °C for later analysis.

### Assays

Lipid biomarkers including low-density lipoprotein cholesterol (LDL), high-density lipoprotein cholesterol (HDL), non-esterified fatty acids (NEFA), TC, and TG were measured using colorimetric assays following the manufacturer’s instruction (Wako Diagnostics, USA). Plasma insulin levels were measured using a mouse insulin ELISA kit (EZRMI-13 K, EMD Millipore Co, Billerica, MA, USA). Adiponectin was measured using a mouse enzyme-linked immunosorbent assays (ELISA) kit (ELM-Adiponectin, RayBiotech, USA). Inflammatory cytokines, including tumor necrosis factor-alpha (TNF-α), interleukin-6 (IL-6), monocyte chemoattractant protein 1 (MCP1), and hormones (leptin and resistin) were measured using a Bio-Plex kit (Biorad, USA). The cytokine assay kit was not sensitive enough to detect the inflammatory cytokines in most of the samples. Therefore, values of these cytokines IL-6, TNF-α, and MCP-1 were excluded.

### Quantitative real-time PCR (qRT-PCR)

RNA was extracted with Trizol reagent (Invitrogen). cDNA was synthesized with 1 µg of total RNA with iScript Reverse Transcription Supermix (Biorad, USA). Gene expression was determined by qRT-PCR (Biorad, USA) using SYBR Green Supermix with gene-specific primer sets real-time PCR using primers as previously used^3,47,48^. The common endogenous reference genes, such as *18 s* rRNA, *Gapdh*, *Actb*, *36B4*, *B2M*, *Tbp*, and *Hprt* were evaluated using the comparative delta-Ct method^49^ as well as M-value, the reference gene expression stability using Bio-Rad CFX Manager 3.2. (Biorad, USA). *Hprt* was most stable among groups, so mRNA levels were normalized to *Hprt* using the cycle threshold (ΔΔCT), and relative fold changes were reported compared to the CON. Primers used for this study are listed in Supplementary Table 2.

### Analysis of myosin-heavy chain content

Left ventricles were homogenized in an extraction buffer (0.3 M NaCl, 0.1 M NaH_2_PO_4_, 0.05 M Na_2_HPO_4_, 0.01 M Na_4_P_2_O_7_, 1 mM MgCl_2,_ and 0.01 M EDTA, pH 6.5) with freshly added 1 mM DTT and protease inhibitor cocktail (Roche) and centrifuged at 4 °C at 12,000 rpm for 10 min. Protein concentration was measured from the supernatant using the Pierce 660 nm assay kit (Pierce, Thermo Fisher Scientific, USA). Homogenized samples (15 µg) were loaded into Acylamide-*N*,*N*'-Diallyl-l-tartardiamide (DATD) gels as previously described^50^. Gel electrophoresis was conducted for 4 h at 16 mA at 4 °C, and the gels were stained with Silver Stain Plus according to the manufacturer's instruction (Biorad, USA). Once stained, all images were taken using the ChemiDoc MP system (Biorad, USA), and the intensity of bands was quantified using Image Lab software version 5.2.1 (https://www.bio-rad.com). Six to seven animals per group were used with two to four technical replicates per animal, and a representative gel image was shown.

### Statistical analyses

All data were presented as a mean ± standard error of the mean (SEM). GraphPad Prism software version 9.0 (https://www.graphpad.com) was used for data analysis of various parameters. Group differences were compared using one-way analysis of variance (ANOVA) or two-way ANOVA followed by Tukey’s multiple comparisons tests. A *p*-value of < 0.05 was considered a significant difference.

## Supplementary Information

Supplementary Information.
