# Supplementary material for: Obesity, not a high fat, high sucrose diet alone, induced glucose intolerance and cardiac dysfunction during pregnancy and postpartum
Source: Sci Rep. 2021 Sep 10;11:18057. doi: 10.1038/s41598-021-97336-x (PMC8433413; doi:10.1038/s41598-021-97336-x)
Supplement: Supplementary file 1 — Supplementary Information. [file 41598_2021_97336_MOESM1_ESM.pdf]

Obesity, not a high fat, high sucrose diet alone, induced insulin resistance and cardiac dysfunction during pregnancy and postpartum

Eunhee Chung[*,1], Kassandra Gonzalez[1], Sarah L. Ullevig[2], John Zhang[1], and Masataka Umeda[1]

[1]Department of Kinesiology and [2]College for Health, Community and Policy, University of Texas at San Antonio, San Antonio, TX.

Ingredient	LFLS (Envigo, TD. 170522)	HFHS (Envigo TD.08811)
Casein	195.0	195.0
L-Cystine	3.0	3.0
Corn starch	409.24	56.86
Maltodextrin	100	60.0
Sucrose	120.0	340.0
Anhydrous Milkfat	37.2	210.0
Cellulose	50.0	50.0
Soybean Oil	32.8	20.0
Mineral mix, AIN-93G-MX (94046)	35.0	43.0
Vitamin Mix, AIN-930VX (94047)	15.0	19.0
Choline Bitartrate	2.75	3.0
TBHQ, antioxidant	0.01	0.04
Green Food Color	0	0.1

Supplementary Table 1. Diet composition fed to mice. The formula was expressed as g/kg to a total of 1,000

Gene	Primer Sequence (5'-3')	
α-MyHC	Forward	ACATTCTTCAGGATTCTCTG
	Reverse	CTCCTTGTCATCAGGCAC
β-MyHC	Forward	TTCCTTACTTGCTACCCTC
	Reverse	CTTCTCAGACTTCCGCAG
PLN	Forward	GTTGTGCCCTTTTTCTACAC
	Reverse	AGAGAGAGCAGATTTGTGG
SERCA2A	Forward	TGTAAGTGGCCAGATTGCTC
	Reverse	CCTAAACAACTGAAGTTAGG
α -skeletal actin	Forward	CGACATCAGGAAGGACCTGTATGCC
	Reverse	AGCCTCGTCGTACTCCTGCTTGG
ANP	Forward	AGGAGAAGATGCCGGTAGAAGA
	Reverse	GCTTCCTCAGTCTGCTCACTCA
BNP	Forward	CAGCTCTTGAAGGACCAAGG
	Reverse	AGAGACCCAGGCAGAGTCAG
Cola1	Forward	GAAACCCGAGGTATGCTTGA
	Reverse	GGGTCCCTCGACTCCTACAT
Col3a1	Forward	CCTGGCTCAAATGGCTCAC
	Reverse	GACCTCGTGTTCCGGGTAT
Col8a1	Forward	CAAGTCCCTCACATGCCTTTG
	Reverse	GCACAGGTGGGATTTCCTTCATA
Ctgf	Forward	TGACCTGGAGGAAAACATTAAGA
	Reverse	AGCCCTGTATGTCTTCACACTG
Ccl2	Forward	GAAGGAATGGGTCCAGACA
	Reverse	ACGGGTCAACTTCACATTCA
Hprt1	Forward	AGCCCCAAAATGGTTAAGGT
	Reverse	CAAGGGCATATCCAACAACA

Supplementary Table 2. Summary of qRT-PCR oligonucleotide primers

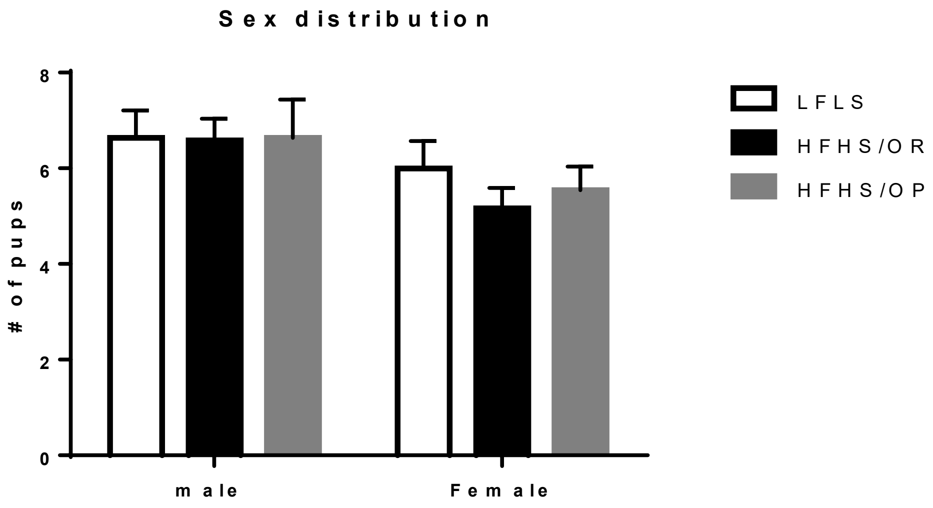

Supplementary Figure 1. Sex distribution. Values are presented as mean ± SEM. CON, white bars; HFHS, black bars; HFHS/OB, gray bars.

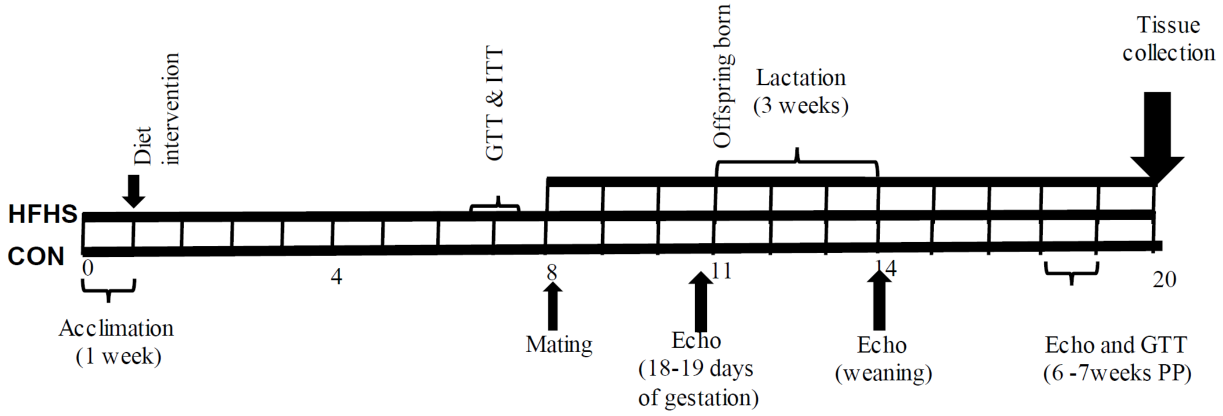

Supplementary Figure 2. Experimental timeline. After one week of acclimatization, dams were fed either HFHS or CON diet. GTT was performed before mating and 6-7 weeks of postpartum, while ITT was measured before mating. Mating began after 8 weeks of diet intervention and echocardiography was performed 18-19 days of gestation (1 to 2 days before parturition), postpartum day 21 (at weaning), and 6 weeks of postpartum. The mice were euthanized at 20-22 weeks of diet intervention, which was 8 weeks of postpartum. CON, control mice fed with a low-fat low sucrose diet; HFHS, mice fed a high-fat high-sucrose diet; GTT, glucose tolerance test; ITT, insulin tolerance test; Echo, echocardiography; PP, postpartum.
